# Oral bacteriome in pediatric patients with malignancies prior to chemotherapy: a pilot study using full-length 16S rRNA sequencing

**DOI:** 10.3389/fcimb.2026.1891342

**Published:** 2026-08-24

**Authors:** Penghui Wu, Xiuchen Guan, Jiajia Zheng, Zheng He, Yunlong Zhao, Jiaqi Wu, Yao Xie

**Affiliations:** 1 Children’s Medical Center, Peking University First Hospital, Beijing, China; 2 First Clinical Division, Peking University School and Hospital of Stomatology & National Center for Stomatology & National Clinical Research Center for Oral Diseases & National Engineering Research Center of Oral Biomaterials and Digital Medical Devices, Beijing, China; 3 Eighth Clinical Division, Peking University School and Hospital of Stomatology & National Center for Stomatology & National Clinical Research Center for Oral Diseases & National Engineering Research Center of Oral Biomaterials and Digital Medical Devices, Beijing, China

**Keywords:** 16S rRNA sequencing, microbial diversity, oral microbiome, pediatric malignancies, pre-chemotherapy

## Abstract

**Objective:**

To characterize the composition, diversity, and ecological features of the oral bacteriome in pediatric patients with malignancies prior to chemotherapy initiation.

**Methods:**

In this prospective pilot study,supragingival plaque samples were collected from 10 pediatric cancer patients prior to the initiation of chemotherapy. Bacterial genomic DNA was extracted from each sample, and the full-length 16S rRNA gene was amplified and sequenced on the PacBio Sequel II platform using circular consensus sequencing (CCS). Raw CCS reads were quality-filtered and denoised into amplicon sequence variants (ASVs) using DADA2, and taxonomic assignment was performed against the SILVA 138 reference database. Alpha diversity was assessed using the Chao1, Shannon, Simpson, and Faith’s phylogenetic diversity (PD whole tree) indices, while beta diversity was evaluated through principal coordinate analysis (PCoA), and non-metric multidimensional scaling (NMDS). Microbial co-occurrence networks were constructed to characterize bacterial interactions, and functional potential was predicted using PICRUSt2, and BugBase.

**Results:**

A total of 614,473 high-quality CCS reads were generated, yielding 1,697 ASVs. Alpha diversity analysis revealed substantial inter-individual variation in microbial richness and diversity among the pediatric cancer patients. The bacterial community was dominated by the phyla *Firmicutes, Proteobacteria, Bacteroidota, Actinobacteriota*. At the genus level, *Streptococcus*, *Prevotella*, *Neisseria*, and *Haemophilus* were the most abundant taxa. Beta diversity analysis revealed distinct clustering patterns, indicating highly individualized microbial profiles. Co-occurrence network analysis identified several keystone taxa and potential pathogenic associations within the supragingival plaque community. Functional prediction indicated that the dominant metabolic pathways were related to amino acid metabolism, carbohydrate metabolism, and membrane transport.

**Conclusion:**

These preliminary findings reveal a taxonomically diverse, highly individualized pre-chemotherapy oral bacteriome, providing foundational baseline profiles to guide future longitudinal investigations of chemotherapy-induced dysbiosis and personalized interventions.

## 1 Introduction

Pediatric malignancies represent a leading cause of disease-related mortality among children worldwide, with hematologic malignancies—predominantly leukemias and lymphomas—accounting for approximately 40% of all childhood cancers (Steliarova-Foucher et al., 2017; GBD 2017 Childhood Cancer Collaborators, 2019). While chemotherapy remains the cornerstone of treatment for most of these malignancies, the associated myelosuppression and immunosuppression markedly increase vulnerability to a wide range of infectious complications (Lehrnbecher et al., 2017). Among these, oral complications—including mucositis, opportunistic infections, and dysbiosis-related systemic infections—are especially common and contribute substantially to treatment-related morbidity, dose reductions or delays, and impaired quality of life (Ribeiro et al., 2017; Patel et al., 2021).

The oral cavity harbors one of the most diverse microbial communities in the human body, encompassing more than 700 bacterial species that coexist in a complex ecological equilibrium with the host immune system (Yamashita and Takeshita, 2017). This oral microbiome plays a critical role in maintaining mucosal barrier integrity, preventing colonization by exogenous pathogens, and modulating both local and systemic immune responses (Sedghi et al., 2021). Under immunocompetent conditions, commensal bacteria competitively inhibit pathogen colonization through niche occupation, production of antimicrobial compounds (e.g. bacteriocins and hydrogen peroxide), and maintenance of a stable microenvironment (Marsh, 2018). However, chemotherapy-induced disruption of this equilibrium—driven by direct cytotoxic effects on the oral epithelium, immunosuppression, and alterations in salivary flow and composition—can precipitate dysbiosis, promoting pathobiont overgrowth and translocation into the systemic circulation (Laheij et al., 2012; Vasconcelos et al., 2016).

Emerging evidence indicates that the pre-treatment (baseline) composition of the oral microbiome may influence susceptibility to chemotherapy-induced oral complications and bloodstream infections (Stoma et al., 2021; McMahon et al., 2023). Characterization of this baseline state is therefore essential for elucidating how chemotherapy perturbs microbial communities and for identifying microbiome-derived biomarkers predictive of complication risk. Third generation long-read platforms—exemplified by the PacBio Sequel II system employing single-molecule real-time (SMRT) circular consensus sequencing—generate high-fidelity, full-length 16S rRNA gene reads that substantially improve species-level discrimination (Zhang et al., 2023). Comparative studies in human microbiome samples, including saliva and gut communities, have demonstrated that full-length 16S rRNA sequencing recovers taxa and ecological features obscured by short-read approaches and yields more accurate taxonomic assignment (Matsuo et al., 2021; González et al., 2025). Applying this methodology to characterize the oral bacteriome of pediatric cancer patients prior to chemotherapy therefore represents a methodological advancement that may reveal clinically relevant taxonomic and ecological features previously inaccessible with short-read sequencing (Zhang et al., 2024).

The present study was designed as a prospective pilot investigation to characterize the oral bacterial community structure in pediatric patients with malignancies prior to chemotherapy initiation, using PacBio full-length 16S rRNA sequencing. The specific objectives were to: (i) describe the composition and diversity of oral bacteria before chemotherapy; (ii) examine how bacterial communities differ between individuals; (iii) identify interactions among oral bacteria; and (iv) predict the functions of these bacterial communities. These baseline findings will serve as a reference for future studies on how chemotherapy alters the oral microbiome and affects clinical outcomes in children with cancer.

## 2 Materials and methods

### 2.1 Design and participants

This prospective pilot study was conducted at the Children’s Medical Center of Peking University First Hospital from January to March 2024. Ten pediatric patients with newly diagnosed malignancies who were scheduled to undergo systemic chemotherapy were enrolled, comprising 5 males and 5 females with a median age of 8.37 years (range, 2.69 – 14.99 years). The underlying malignancies included leukemia (n=3), lymphoma (n=2), rhabdomyosarcoma (n=2), Wilms tumor (n=1), neuroblastoma (n=1), and germ cell tumor (n=1).

Patients were eligible for inclusion if they met all the following criteria: (i) age between 1 and 16 years; (ii) a pathologically confirmed malignancy with a clinical indication for systemic chemotherapy; (iii) no systemic corticosteroid use within the three months preceding enrollment; and (iv) no exposure to antibiotics or active probiotic supplements within the two weeks preceding enrollment. Patients were excluded if they had (i) concurrent systemic or severe gastrointestinal infections at the time of diagnosis, or (ii) a requirement for specialized dietary regimens due to underlying metabolic or systemic disease.

### 2.2 Ethical approval

Ethical approval for this study was granted by the Ethics Committee of Peking University First Hospital (Approval No. 2024-459-002), and all procedures were performed in accordance with the ethical standards of the Declaration of Helsinki. Written informed consent was obtained from the parents or legal guardians of each participant prior to enrollment.

### 2.3 Sample collection

#### 2.3.1 Oral swab sample collection

Oral swab samples were collected at baseline, immediately prior to the initiation of systemic chemotherapy. Patients were instructed to fast for at least 2 hours prior to sampling. After thoroughly rinsing the oral cavity with sterile saline, samples were obtained from the occlusal, labial, and buccal surfaces of the maxillary and mandibular molars using sterile cotton swabs, with four consecutive strokes applied at each site. The swabs were then immediately transferred into sterile collection tubes and stored at −80 °C until further processing.

#### 2.3.2 DNA extraction and PCR amplification

Total genomic DNA was extracted from oral swab samples using a commercial DNA extraction kit following the manufacturer’s protocol. DNA concentration and purity were assessed by spectrophotometry (NanoDrop One, Thermo Fisher Scientific) and agarose gel electrophoresis.

The full-length 16S rRNA gene was amplified using the universal bacterial primers 27F (5’-AGAGTTTGATCMTGGCTCAG-3’) and 1492R (5’-TACGGYTACCTTGTTACGACTT-3’). PCR amplification was performed in a total volume of 25 μL containing 12.5 μL of 2x PCR Master Mix, 1 μL of each primer (10 μM), 1 μL of template DNA, and 9.5 μL of nuclease-free water. Thermal cycling conditions were: initial denaturation at 95 °C for 5 min, followed by 30 cycles of 95 °C for 30 s, 56 °C for 30 s, and 72 °C for 90 s, with a final extension at 72 °C for 10 min. Each sample was amplified in triplicate, and the resulting amplicons were pooled and purified using Agencourt AMPure XP beads.

#### 2.3.3 Library preparation and sequencing

Purified amplicons were quantified using the Qubit dsDNA HS Assay Kit (Thermo Fisher Scientific) and pooled at equimolar concentrations. SMRTbell libraries were prepared according to the PacBio protocol and sequenced on the PacBio Sequel II platform using Circular Consensus Sequencing (CCS) mode with a read quality threshold of Q20.

### 2.4 Bioinformatic analysis

#### 2.4.1 Data preprocessing

Raw sequencing data were processed using a standardized bioinformatics pipeline. Circular consensus sequence (CCS) reads were generated with SMRT Link v9.0 (Pacific Biosciences) using a minimum of 3 passes and a minimum predicted accuracy of 0.99. Barcodes were identified using lima v1.7.0, after which primer sequences were trimmed and reads were length-filtered with cutadapt v1.9.1 to retain sequences of 1300–1700 bp. Denoising and amplicon sequence variant (ASV) generation were performed using DADA2 as implemented in QIIME2 v2020.6 (Callahan et al., 2016), with chimeras removed using the consensus method. Representative ASV sequences were taxonomically classified with a naive Bayes classifier trained on the SILVA 138 database (Quast et al., 2013), applying a confidence threshold of 0.8. Species-level annotations were further refined by BLAST+ searches against the SILVA reference database, using minimum thresholds of 97% identity and 90% query coverage.

#### 2.4.2 Diversity analysis

Alpha diversity was characterized using a suite of complementary indices, namely the number of observed features, Chao1 richness, the Shannon and Simpson indices, and Faith’s phylogenetic diversity (PD), all of which were computed with the q2-diversity plugin implemented in QIIME2 (Bolyen et al., 2019). Prior to diversity estimation, samples were rarefied to an even sequencing depth, which was selected based on rarefaction curves to ensure that the sampling effort adequately captured the community diversity present in each sample. Beta diversity was evaluated using Bray–Curtis dissimilarities derived from the rarefied ASV table (vegdist function, vegan package v2.6-4, R v4.2.1). Inter-individual variation in microbial composition was visualized using principal coordinate analysis (PCoA), alongside non-metric multidimensional scaling (NMDS; metaMDS function) as a complementary ordination. The goodness of fit for the NMDS was assessed via the stress value (acceptable if <0.2, excellent if <0.05). Given the descriptive, single-group design of this pilot study (n=10), beta diversity was assessed visually without formal between-group statistical testing.

#### 2.4.3 Co-occurrence network analysis

Microbial co-occurrence networks were constructed at the genus level based on pairwise Spearman’s rank correlations. Only robust associations, defined as those with an absolute correlation coefficient |r| > 0.1 and a *P* < 0.05, were retained for network assembly. Networks were visualized in Gephi, and topological properties, including modularity, the clustering coefficient, and node centrality, were calculated to describe network architecture. To identify putative keystone taxa, the within-module connectivity (Zi) and among-module connectivity (Pi) of each node were determined, and taxa were classified according to their topological roles following the thresholds described by Olesen et al. (2007).

#### 2.4.4 Functional prediction

The metabolic potential of the microbial communities was inferred from the 16S rRNA gene data using PICRUSt2, which was applied to predict Kyoto Encyclopedia of Genes and Genomes (KEGG) orthologs and the associated metabolic pathways. To complement these predictions at the phenotypic level, BugBase was used to estimate community phenotypes, including oxygen tolerance and potential pathogenicity.

### 2.5 Statistical analysis

All statistical analyses were performed in R (v4.2.1). Alpha diversity metrics were compared between groups using the Wilcoxon rank-sum test (two-group comparisons) or the Kruskal-Wallis test (comparisons of three or more groups). Differences in beta diversity among groups were assessed by permutational multivariate analysis of variance (PERMANOVA) based on Bray-Curtis dissimilarities with 999 permutations, implemented in the vegan package (v2.6-4). Unless otherwise specified, statistical significance was defined as *P* < 0.05, and an adjusted *P* < 0.05 was applied for differential abundance testing.

## 3 Results

### 3.1 Demographic characteristics of the participants

A total of 10 pediatric patients with malignancies were enrolled in this study, comprising 5 males (50%) and 5 females (50%), yielding a male-to-female ratio of 1:1. The median age at diagnosis was 8.37 years (range: 2.69–14.99 years; mean ± SD: 8.61 ± 5.09 years) (**Table 1**). Of note, paired ITS sequencing data from the same cohort have been analyzed separately to characterize the oral mycobiome and are reported in a companion manuscript; the present study focuses exclusively on the bacterial community profile derived from 16S rRNA gene sequencing.

**Table 1:** Demographic profile of the participants.

Characteristic	Category	n
Sex	Male	5
	Female	5
Age	0–3 years	1
	3–6 years	4
	6–12 years	2
	12–16 years	3
	Median (range)	8.37 (2.69–14.99) years
Tumor type	Leukemia	3
	Lymphoma	2
	Rhabdomyosarcoma	2
	Wilms tumor	1
	Neuroblastoma	1
	Germ cell tumor	1

### 3.2 Sequencing data quality and processing

A total of 614,473 high-quality circular consensus sequencing (CCS) reads were generated from 10 supragingival plaque samples, averaging 61,447 reads per sample (range: 55,039–68,490). After barcode demultiplexing, primer trimming, and length filtering, a mean of 58,410 clean reads per sample was retained. DADA2 denoising and chimera removal yielded a total of 1,697 amplicon sequence variants (ASVs) (**Table 2**).

**Table 2:** Summary of PacBio CCS sequencing data and ASV statistics for 10 supragingival plaque samples.

Sample	Tumor type	Raw CCS	Clean CCS	Non-chimeric		ASVs	Species
M1	Leukemia	58,648	58,592	49,315		164	71
M2	Wilms Tumor	58,434	58,356	49,285		149	76
M3	Neuroblastoma	63,972	63,961	54,103		298	109
M4	Rhabdomyosarcoma	57,128	57,110	54,808		185	95
M5	Germ cell tumor	64,185	64,177	56,828		188	71
M6	Leukemia	55,039	55,004	50,269		142	74
M7	Leukemia	65,544	65,537	62,625		211	125
M8	Lymphoma	62,185	62,176	58,445		208	79
M9	Rhabdomyosarcoma	60,848	60,720	48,443		174	72
M10	Lymphoma	68,490	68,442	65,091		377	177
**Mean**		**61,447**	**61,455**	**54,921**		**210**	**95**

The bold values show that the average number of reads per sample is 61,447 (range: 55,039–68,490), which indicates the sequencing depth.

### 3.3 Taxonomic composition

**Figure 1:**
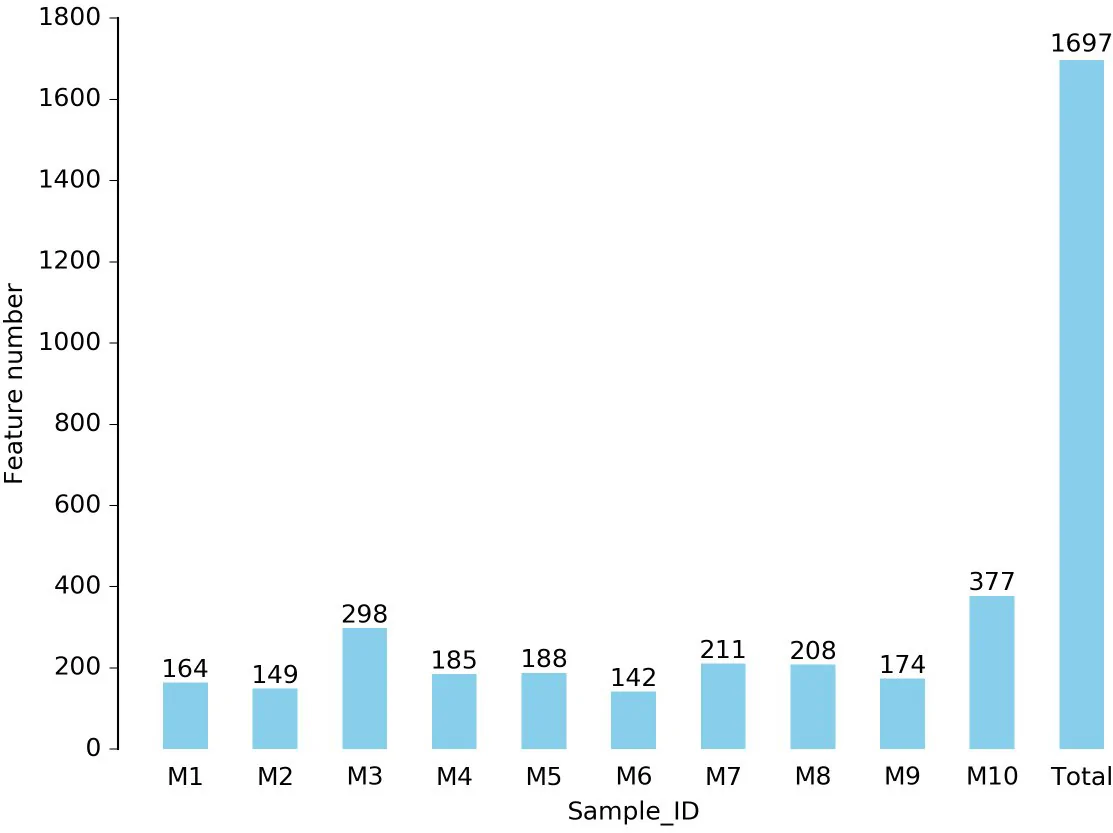
Distribution of feature counts across individual samples.

The number of ASVs per sample ranged from 142 to 377 (mean: 209.6 ± 76.4), reflecting substantial inter-individual variation (**Figure 1**). Taxonomic annotation rates were high across all ranks: 99.98% of reads were classified at the phylum level, 99.92% at the class level, 99.76% at the order level, 99.15% at the family level, 93.08% at the genus level, and 64.24% at the species level. This species-level annotation rate substantially exceeds the 30–50% typically reported for short-read studies, underscoring the improved taxonomic resolution afforded by full-length 16S rRNA gene sequencing.

**Figure 2:**
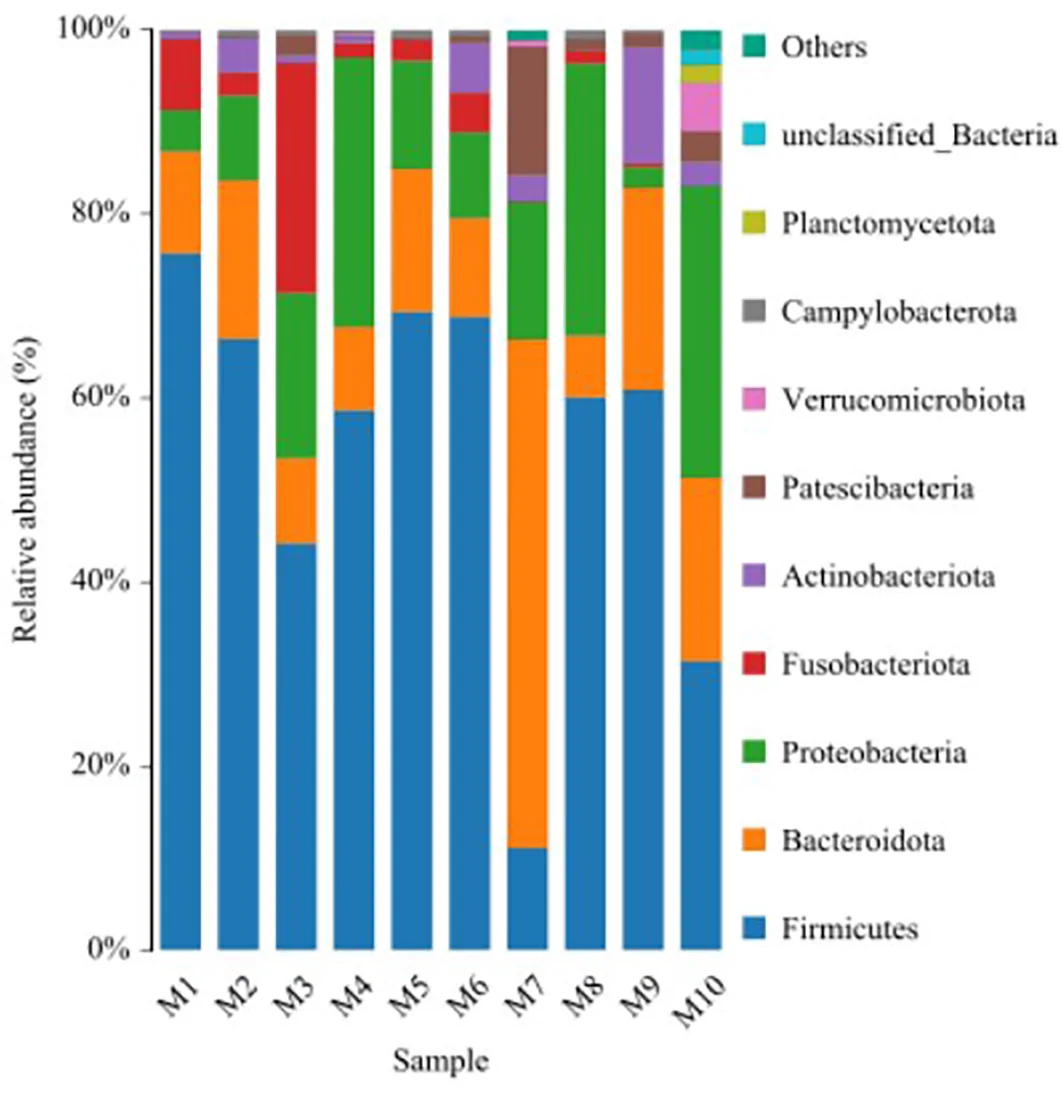
Phylum-level relative abundance of the bacterial community across individual samples.

To characterize the structure of the bacterial communities across the ten samples (M1–M10), the relative abundances of the dominant phyla were assessed (**Figure 2**). In total, the communities were composed predominantly of *Firmicutes, Bacteroidota*, and *Proteobacteria*, which together constituted the large majority of the sequences in nearly all samples. Eight additional taxa—*Fusobacteriota, Patescibacteria, Verrucomicrobiota, Campylobacterota, Planctomycetota*, unclassified Bacteria, and a residual “Others” category—were detected at comparatively lower relative abundances.

**Figure 3:**
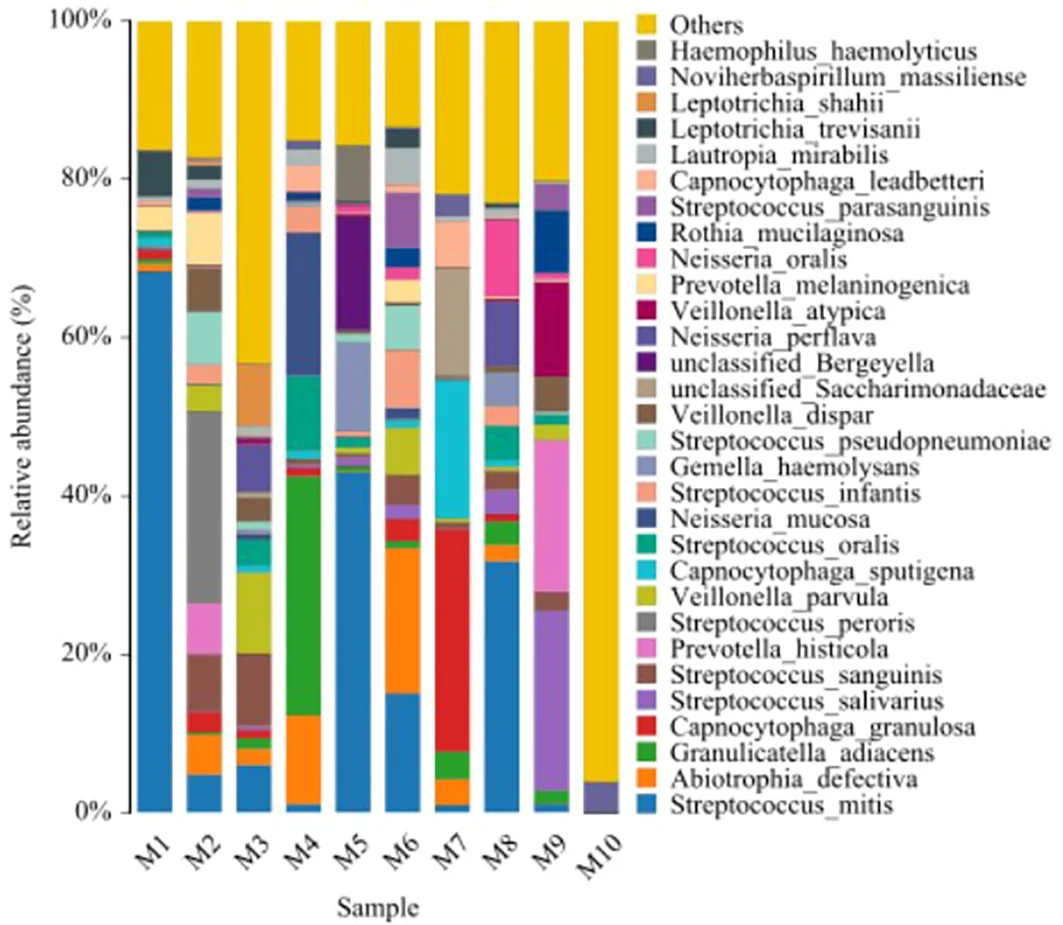
Relative abundance of the most prevalent bacterial species across samples M1–M10.

To resolve the bacterial communities at finer taxonomic resolution, the relative abundances of the most prevalent species were examined across the ten samples (M1–M10) (**Figure 3**). The communities were characterized by a high degree of inter-sample heterogeneity, and a substantial proportion of the sequences in several samples fell within the “Others” category, indicating considerable taxonomic diversity beyond the most abundant species displayed.

*Streptococcus mitis* was the single most dominant species in several samples and exhibited pronounced variation among individuals. It reached its highest relative abundance in M1 (approximately 68%), where it overwhelmingly dominated the community, and was also a major constituent of M5 (approximately 43%), M8 (approximately 32%), and M6 (approximately 15%). In contrast, S. mitis was markedly depleted in M2, M9, and M10, where it contributed only minor fractions of the community.

Beyond S. mitis, several additional species contributed prominently to individual samples in a markedly sample-specific manner. Abiotrophia defectiva was notably enriched in M4 and M7, while *Granulicatella adiacens* was particularly abundant in M4, where it represented one of the principal constituents. *Streptococcus salivarius* showed elevated representation in M2, and *Streptococcus sanguinis* contributed appreciably to M2 and M3.

These results demonstrate that, while *Streptococcus mitis* constituted the predominant species in several samples (most M1), the species-level composition of the bacterial communities was highly variable across individuals. The pronounced enrichment of distinct taxa—such as *Abiotrophia defectiva* and *Granulicatella adiacens* in M4 and M7, *Prevotella histicola* in M9, and the predominance of unclassified or low-abundance taxa in M10—underscores the substantial inter-individual heterogeneity in community structure at the species level.

### 3.4 Alpha diversity analysis

Alpha diversity indices varied considerably across the 10 patients, reflecting substantial inter-individual differences in community richness, evenness, and phylogenetic diversity (**Table 3**).

**Figure 4:**
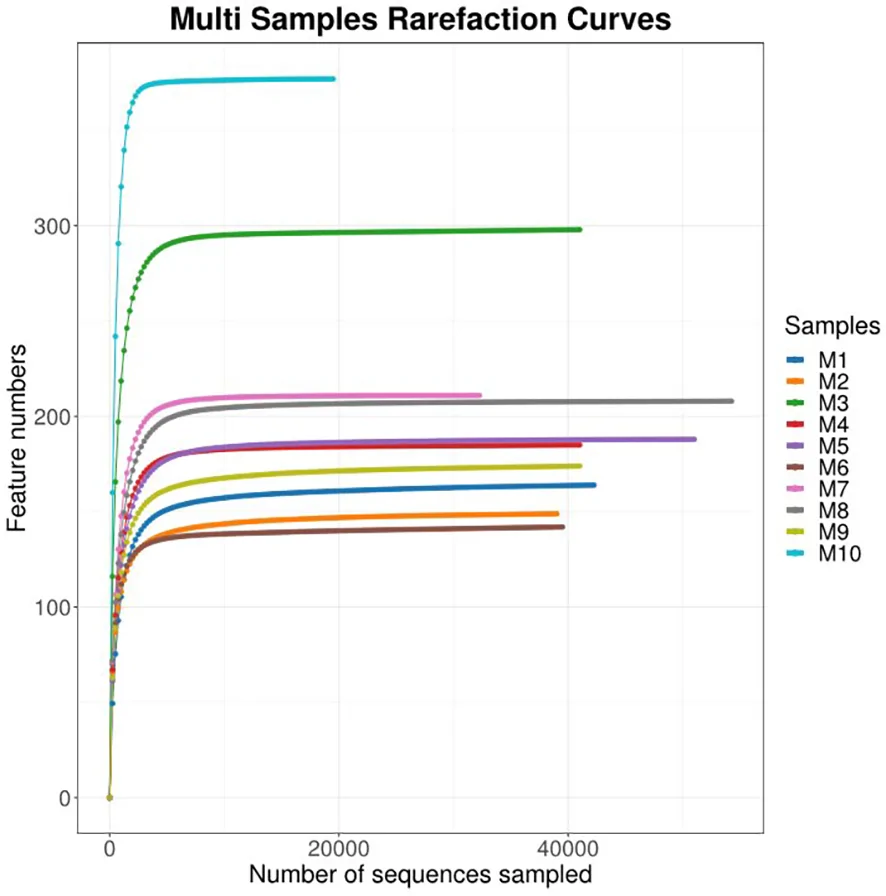
Rarefaction curves of oral bacteriome communities in pediatric patients with malignancies.

Rarefaction curves for all samples approached saturation, indicating adequate sequencing depth, and the species accumulation curve plateaued as sample size increased, suggesting that the 10 samples captured most of the community diversity (**Figure 4**).

Alpha diversity analysis revealed substantial inter-individual variation in microbial community structure among the 10 pediatric patients. Chao1 richness estimates ranged from 143.0 to 377.0 (mean ± SD: 200.9 ± 72.3), indicating considerable differences in species richness among individuals. Shannon diversity indices ranged from 3.67 to 8.13 (5.63 ± 1.29), and Simpson indices ranged from 0.78 to 0.99 (0.93 ± 0.06). Sample M10 exhibited the highest diversity across all metrics (Shannon, 8.13; Simpson, 0.99; Chao1, 377.0). Sample M1 showed the lowest overall diversity based on Shannon (3.67) and Simpson (0.78) indices, while M6 exhibited the lowest species richness (Chao1, 143.0). Phylogenetic diversity (PD whole tree) ranged from 4.95 to 9.40 (6.19 ± 1.30), reflecting differences in both taxonomic richness and evolutionary relatedness among communities. Good’s coverage exceeded 0.999 for all samples, confirming that the sequencing depth was sufficient to capture the vast majority of microbial diversity present (**Table 3**).

### 3.5 Beta diversity analysis

**Figure 5:**
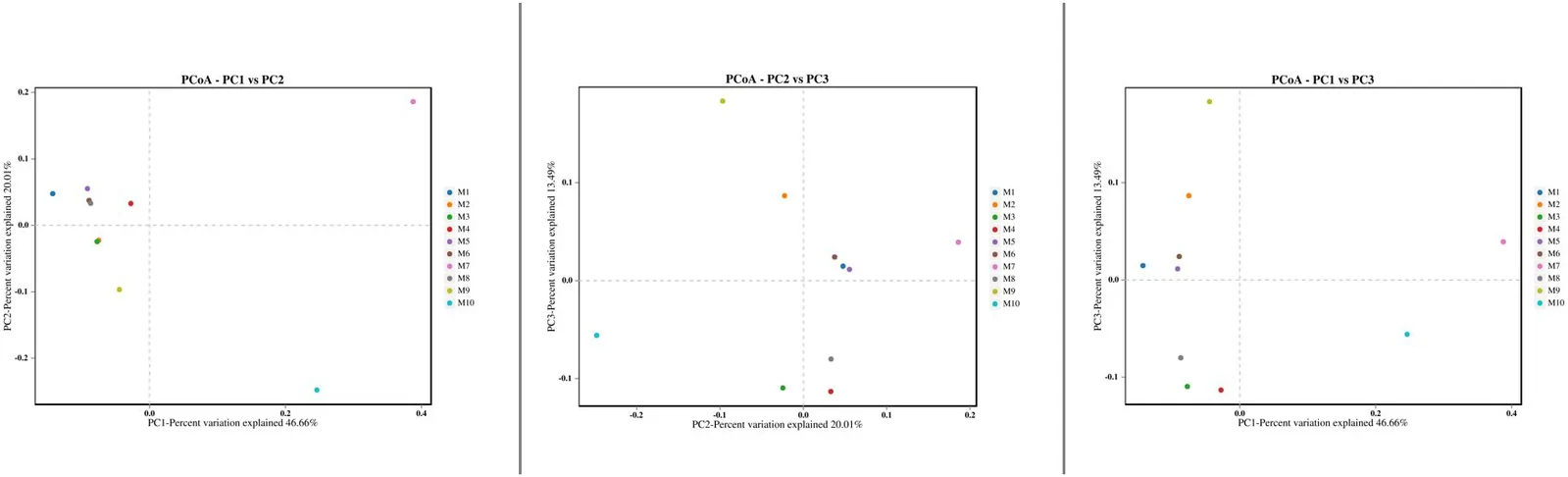
Principal coordinate analysis (PCoA) of microbial community composition across samples (M1–M10).

Beta diversity analysis revealed pronounced differences in bacterial community composition and structure among the ten patients. To explore inter-individual variation in microbial community composition, we performed Principal Coordinate Analysis (PCoA) and visualized the relationships among the ten samples (M1–M10) across the first three principal coordinate axes (**Figure 5**). The first three axes together accounted for 80.35% of the total variation, with PC1, PC2, and PC3 explaining 46.66%, 20.10%, and 13.59% of the variance, respectively. Across all three pairwise projections, the samples did not segregate into clearly defined clusters. Instead, the ordination was dominated by a small number of dispersed outlier samples (notably M5, M9, and M10), while the remaining individuals broadly overlapped. This pattern is consistent with high inter-individual variability in microbial community structure within this small cohort.

**Figure 6:**
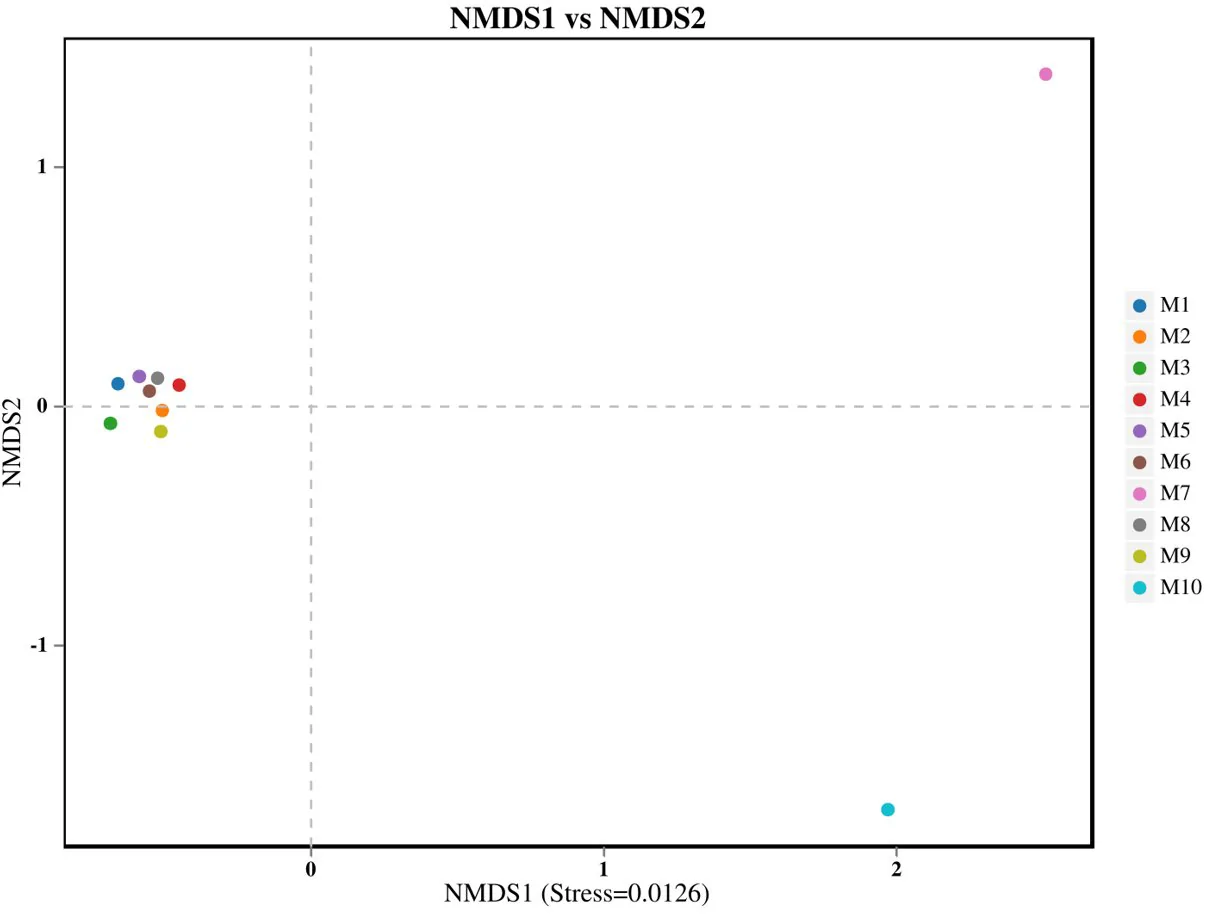
Non-metric multidimensional scaling (NMDS) ordination of microbial community composition across samples.

To further examine the dissimilarity in microbial community composition among the ten samples (M1–M10), non-metric multidimensional scaling (NMDS) was performed to visualize these relationships in two dimensions (**Figure 6**). The ordination achieved a very low stress value of 0.0126, well below the conventional threshold of 0.2 (and even the more stringent threshold of 0.05), indicating an excellent two-dimensional representation of the underlying multidimensional distances between samples. The NMDS analysis corroborated the patterns observed in the PCoA ordination: most samples shared a broadly similar community structure, whereas a small number of samples (notably M7 and M10) exhibited distinctly divergent compositions and accounted for the majority of the between-sample variation. Given the small sample size (n = 10), these results should be regarded as descriptive and exploratory.

### 3.6 Co-occurrence network analysis

**Figure 7:**
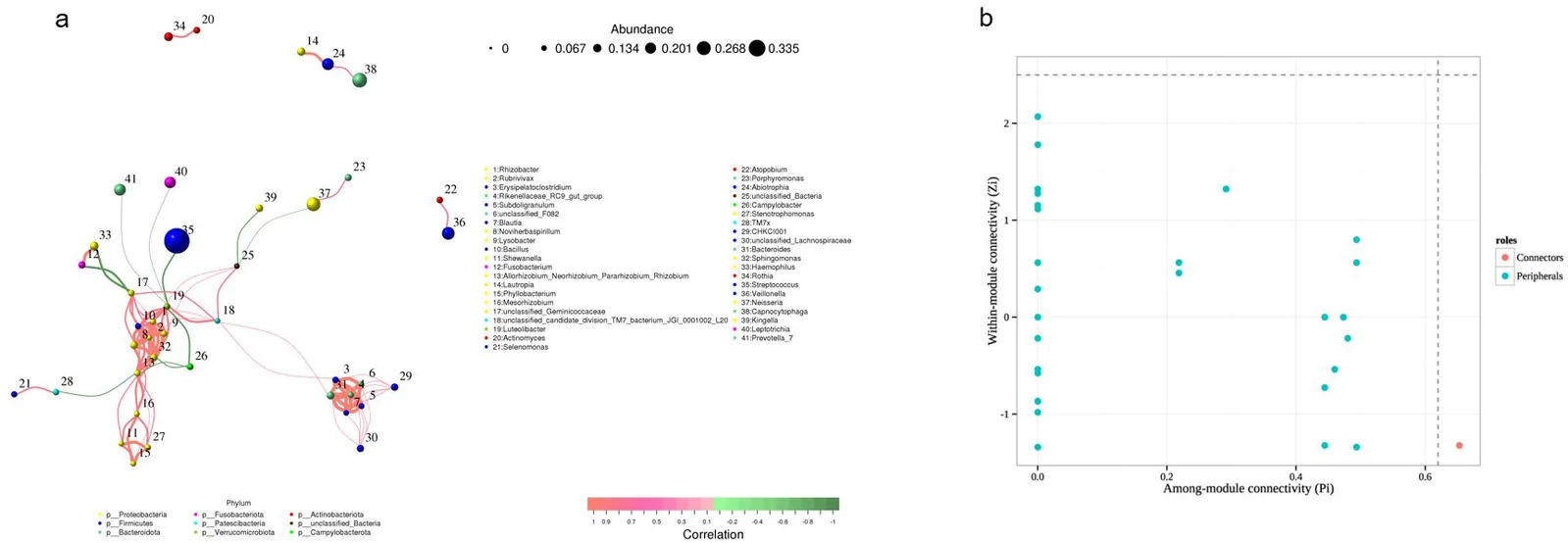
**(a)** Co-occurrence network of the dominant microbial taxa. **(b)** Zi–Pi plot showing the topological roles of nodes within the co-occurrence network.

To explore the potential ecological interactions and the structural organization of the microbial community, we constructed a co-occurrence network based on significant pairwise correlations among the dominant taxa (**Figure 7a**). The resulting network consisted of 41 nodes, each corresponding to a distinct taxon, with node colour denoting phylum-level affiliation and node size scaled to relative abundance (ranging from rare taxa to highly abundant members at ~0.335). Edges represented statistically significant correlations between taxa, with positive correlations shown in red and negative correlations in green; the intensity of edge colour reflected the magnitude of the correlation coefficient (–1 to +1).

The network exhibited a clearly modular topology, comprising several densely interconnected sub-clusters together with several peripheral and loosely associated nodes. The most prominent module, located in the lower-left region of the network, was dominated by members of the phyla *Proteobacteria* and *Firmicutes* and was characterized by a high density of intra-modular connections. Within these tightly linked clusters, positive correlations (red edges) predominated, suggesting co-occurrence or potential cooperative relationships among the constituent taxa. In contrast, negative correlations (green edges) were comparatively sparse and tended to connect otherwise distinct modules. Several taxa (e.g., nodes 14, 20, 24, 34, and 38) formed small satellite groups that were largely disconnected from the central network core, indicating limited interaction with the broader community.

To classify the topological role of each taxon, nodes were categorized based on their within-module connectivity (Zi) and among-module connectivity (Pi) (**Figure 7b**). Using the standard thresholds (Zi = 2.5; Pi = 0.62), most nodes were classified as peripheral nodes (cyan), indicating that the majority of taxa interacted mainly within their own modules and contributed little to inter-module connectivity.

No module hubs or network hubs were detected, and only one taxon was identified as a connector (red), serving as the main bridge between modules. The predominance of peripheral nodes and the lack of hubs suggest that the network was not driven by dominant keystone taxa but was instead organized into relatively independent modules linked by a few bridging taxa.

### 3.7 Functional prediction

**Figure 8:**
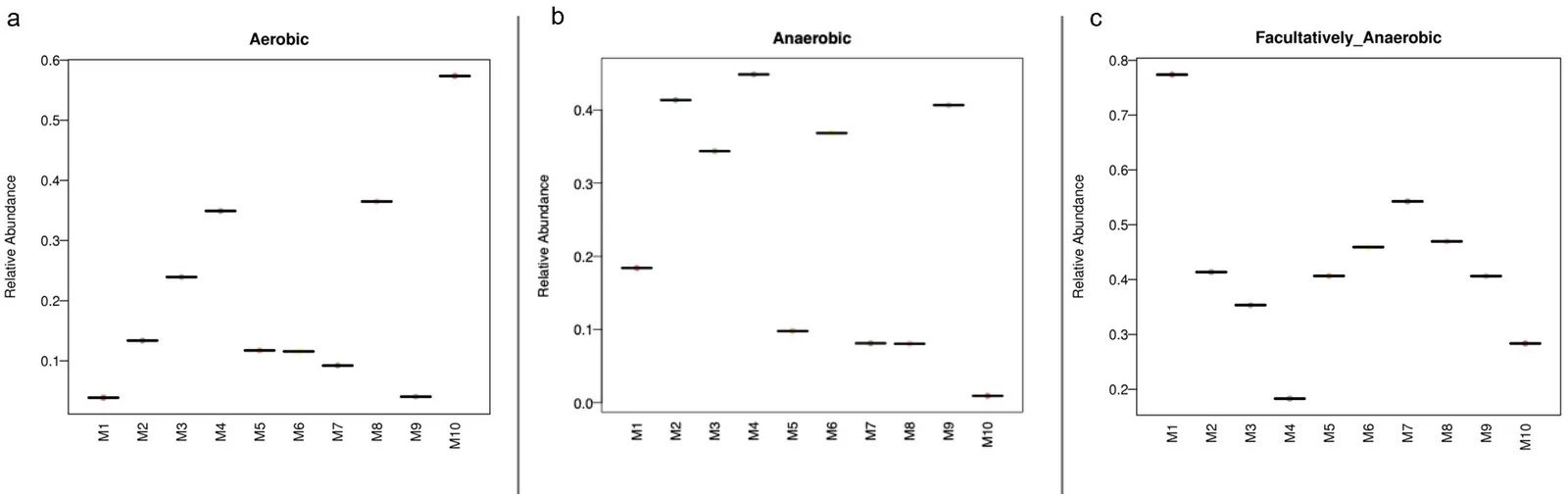
Relative abundance of microbial oxygen-utilization phenotypes across supragingival plaque samples of pediatric tumor patients: **(a)** aerobic; **(b)** anaerobic; **(c)** facultatively anaerobic.

To explore the functional composition of the microbial communities, the dominant taxa were classified according to their oxygen-utilization phenotype, and the relative abundances of aerobic, anaerobic, and facultatively anaerobic bacteria were compared across the ten patient samples (M1–M10). Facultatively anaerobic bacteria were the predominant phenotype across most samples, with relative abundances ranging from approximately 0.18 (M4) to 0.77 (M1) (**Figure 8c**). The highest proportion was observed in a Leukemia sample (M1), whereas the lowest occurred in the rhabdomyosarcoma sample (M4),as shown in **Figure 8c**.

Analysis of the microbial oxygen-utilization phenotypes revealed substantial inter-sample variability in the relative abundance of aerobic microorganisms across the ten supragingival plaque samples (M1 to M10) from pediatric tumor patients (**Figure 8a**). The relative abundance of the aerobic phenotype ranged widely, from less than 0.05 to nearly 0.60. Sample M10 exhibited the highest proportion of aerobic bacteria, followed by samples M8 and M4. Sample M1 and M9 displayed the lowest relative abundances, both falling below 0.05. These findings highlight a marked heterogeneity in the aerobic microbial composition within the plaque of this pediatric patient cohort.

Aerobic bacteria generally constituted the smallest fraction of the community across samples (**Figure 8a**), with relative abundances below 0.40 in nine of the ten samples. A notable exception was the lymphoma sample M10, in which aerobic bacteria dominated the community (~0.57), corresponding to its markedly reduced anaerobic fraction.

**Figure 9:**
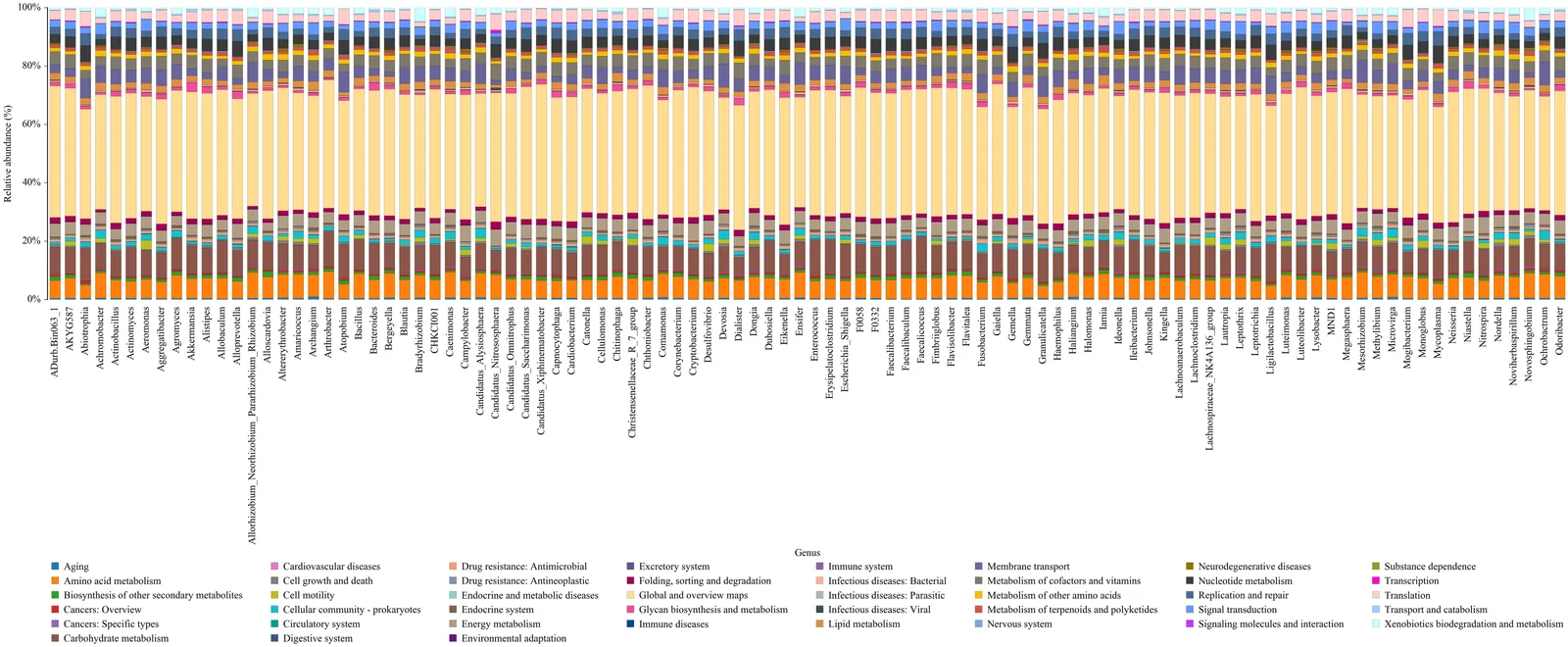
Predicted KEGG functional profiles of the tumor-associated microbiota. Stacked bar plots show the relative abundances (%) of KEGG level-2 functional categories predicted for each bacterial genus.

KEGG functional categories (level 2) were predicted for each bacterial genus and visualized as relative abundances (**Figure 9**). The profiles were broadly conserved across all genera, indicating strong functional redundancy within the tumor-associated microbiota.

Metabolism-related pathways dominated the functional landscape. Carbohydrate metabolism and amino acid metabolism were the most abundant categories across nearly all genera, followed by metabolism of cofactors and vitamins, energy metabolism, and nucleotide metabolism.

Functions related to xenobiotics biodegradation, secondary metabolite biosynthesis, and disease pathways (e.g., antimicrobial drug resistance and infectious diseases) were detected consistently but at lower relative abundances. The functional profiles were dominated by core metabolic and information-processing pathways and were largely uniform across genera, suggesting functional redundancy despite taxonomic diversity.

## 4 Discussion

This pilot study is among the first to apply full-length 16S rRNA gene sequencing on the PacBio platform to characterize the oral bacteriome of pediatric patients with malignancies prior to chemotherapy. Using DADA2 for amplicon sequence variant (ASV) inference and the near-species-level resolution provided by PacBio circular consensus sequencing (CCS), we characterized the taxonomic composition, diversity, ecological interactions, and functional potential of the pre-chemotherapy supragingival plaque microbiome in this clinically vulnerable population.

Hierarchical profiling at both the phylum and species levels revealed a microbial architecture consistent with the established structure of the supragingival plaque microbiome, while also highlighting substantial inter-individual variability in community composition. At the phylum level, the communities were dominated by *Firmicutes, Proteobacteria, Bacteroidota, Fusobacteriota*, and *Actinobacteriota*, which together accounted for most sequences across nearly all ten samples (M1–M10). This pattern aligns with findings from the Human Microbiome Project (HMP) and other large-scale oral microbiome studies, which have consistently identified these five phyla as the predominant constituents of healthy supragingival plaque in both adult and pediatric populations (Human Microbiome Project Consortium, 2012; Segata et al., 2012; Kunath et al., 2024). The conservation of this core phylum-level architecture across diverse cohorts suggests that the broad ecological framework of the supragingival microbiome remains largely intact even in the context of underlying systemic malignancy, prior to the initiation of cytotoxic therapy.

Species-level profiling revealed a high degree of inter-sample heterogeneity (**Figure 3**). *Streptococcus* was the single most dominant species in several samples, yet its relative abundance varied dramatically—from approximately 68% in M1 to near-absence in M2, M9, and M10. This pronounced variation likely contributes substantially to the beta diversity patterns observed in our PCoA and NMDS analyses. Beyond S. mitis, several species showed sample-specific enrichment: Abiotrophia defectiva and Granulicatella adiacens were notably abundant in M4 and M7, Streptococcus salivarius was elevated in M2, and Prevotella histicola characterized M9. The predominance of unclassified or low-abundance taxa in M10 contributed to its exceptionally high Shannon diversity index (8.13) and ASV count (377), making it the most taxonomically diverse sample in the cohort.

These findings are consistent with the known high inter-individual variability of the pediatric oral microbiome, which is shaped by a complex interplay of host genetics, diet, oral hygiene practices, and antimicrobial exposure (Freire et al., 2020). In the context of pediatric malignancy, additional factors—including tumor-related immune perturbations, pre-diagnostic antibiotic use, and variable nutritional status—may further contribute to the individualized community profiles observed. However, given the absence of a healthy control group and the small sample size, we cannot determine whether the degree of heterogeneity observed here exceeds that of healthy children of comparable age.

Alpha diversity analysis revealed substantial inter-individual variation in community richness, evenness, and phylogenetic diversity. Chao1 richness estimates ranged from 143.0 (M6) to 377.0 (M10), and Shannon diversity indices ranged from 3.67 (M1) to 8.13 (M10) (**Table 3**). Sample M10 exhibited the highest diversity across all metrics, a pattern attributable to a more even distribution of species abundances relative to the other samples. In contrast, M1 showed the lowest diversity (Shannon, 3.67), reflecting the overwhelming dominance of *Streptococcus mitis*(~68% relative abundance). Rarefaction curves approached saturation for all samples (**Figure 4**), and Good’s coverage exceeded 0.999 in every case, confirming that the sequencing depth was sufficient to capture most of the microbial diversity present (Hughes et al., 2001).

**Table 3:** Alpha diversity indices of the oral bacteriome across individual samples.

Sample	ASVs	Chao1	Shannon	Simpson	PD tree	Coverage
M1	164	166.1	3.67	0.78	7.02	0.9999
M2	149	150.4	5.29	0.93	5.32	0.9999
M3	298	301.0	7.11	0.99	7.07	0.9999
M4	185	185.0	5.36	0.94	5.44	1.0000
M5	188	188.0	4.68	0.87	4.95	1.0000
M6	142	143.0	5.77	0.97	5.97	0.9999
M7	211	211.0	5.17	0.92	6.42	1.0000
M8	208	208.0	5.52	0.94	5.90	1.0000
M9	174	180.0	5.21	0.94	5.42	0.9999
M10	377	377.0	8.13	0.99	9.40	1.0000

Beta diversity analysis revealed pronounced differences in bacterial community composition among the ten patients. Principal coordinates analysis (PCoA) of Bray–Curtis dissimilarities showed that the first three principal coordinates collectively explained 80.35% of the total variation (PC1, 46.66%; PC2, 20.10%; PC3, 13.59%) (**Figure 5**). The ordination was dominated by two clear outlier samples—M7 and M10—whereas the remaining eight samples formed a relatively compact cluster with partial overlap. This pattern indicates that, although most patients shared a broadly similar community structure, a subset harbored distinctly divergent microbiomes.

Non-metric multidimensional scaling (NMDS) corroborated the PCoA findings, achieving an exceptionally low stress value of 0.0126—well below the conventional threshold of 0.2 and the more stringent criterion of 0.05—indicating an excellent two-dimensional representation of the underlying compositional dissimilarities (**Figure 6**). M7 and M10 again emerged as the most divergent samples. The pronounced separation of M10 along NMDS1 may reflect its unique aerobic-dominant phenotype and high taxonomic diversity, whereas the isolated position of M7 along the same axis may be driven by its distinctive phylum-level composition, characterized by a relative depletion of *Firmicutes* and an enrichment of *Bacteroidota*. The remaining eight samples clustered together without evident segregation by tumor type, reinforcing the observation that, in this cohort, inter-individual variability outweighs tumor-type effects in shaping microbial assemblages. Given the small sample size (n = 10) and the unbalanced distribution of tumor types (1–3 patients per category), these beta diversity patterns should be regarded as descriptive and exploratory rather than as evidence of tumor-specific microbiome signatures (Knight et al., 2018).

Co-occurrence network analysis based on genus-level Spearman correlations revealed an ecological architecture of moderate complexity, comprising 41 nodes connected by multiple edges that linked taxa across different phyla (**Figure 7a**). The network displayed a clearly modular topology, organized into several densely interconnected sub-clusters. The most prominent module was dominated by members of the *Proteobacteria* and *Firmicutes* and was characterized by a high density of positive intra-modular correlations, suggesting potential metabolic cooperation or shared environmental preferences among the constituent taxa (Coyte et al., 2015; Layeghifard et al., 2017). In contrast, negative correlations were comparatively sparse and tended to connect otherwise distinct modules, possibly reflecting competitive exclusion or divergent niche requirements (Faust et al., 2012; Kost et al., 2023).

Zi–Pi plot analysis classified the vast majority of nodes as peripheral taxa with limited inter-module connectivity (**Figure 7b**). No module hubs or network hubs were identified, and only a single taxon was classified as a connector, serving as a bridge between modules (Guimerà and Nunes Amaral, 2005). The predominance of peripheral nodes and the absence of dominant keystone taxa indicate that the ecological organization of the pre-chemotherapy oral microbiome in this cohort is characterized by relatively independent functional modules linked by a few bridging species (Banerjee et al., 2018). This distributed architecture may have implications for community resilience: the scarcity of highly connected keystone taxa could render the microbiome more susceptible to cascading compositional shifts following chemotherapy-induced perturbation, as the loss of a bridging connector may disrupt inter-module communication and facilitate pathogen overgrowth (Berry and Widder, 2014). Nevertheless, this interpretation remains speculative given the small sample size and the inherently correlational nature of network analysis (Röttjers and Faust, 2018).

The positive correlations observed between *Prevotella* and *Veillonella*—both anaerobic or microaerophilic—and between *Neisseria* and *Haemophilus*—both fastidious, capnophilic Gram-negative organisms—are ecologically coherent, reflecting shared environmental preferences and overlapping physiological requirements (Socransky et al., 1998; Harvey et al., 2001). Such co-occurrence patterns are consistent with documented metabolic interdependencies within the oral cavity, most notably the well-characterized cross-feeding relationship in which *Veillonella* utilizes lactate generated by neighboring fermentative taxa such as *Streptococcus* (Mashima and Nakazawa, 2014). The negative correlation between *Prevotella* and *Streptococcus* may reflect competitive exclusion, as these genera frequently compete for overlapping niches and substrates within the oral microbial community (Kolenbrander et al., 2010).

BugBase prediction of oxygen-utilization phenotypes revealed substantial inter-individual variation (**Figure 8**). Facultatively anaerobic bacteria represented the predominant phenotype across most samples, with relative abundances ranging from approximately 0.18 (M4) to 0.77 (M1). Anaerobic bacteria exhibited marked variability, reaching their highest proportions in M2, M3, and M4 (~0.34–0.45) and their lowest in M10 (~0.01). Aerobic bacteria generally constituted the smallest fraction across samples, with a notable exception in M10, in which aerobic taxa dominated the community (~0.57). The aerobic-dominant profile of M10, together with its exceptionally high taxonomic diversity, suggests a potentially distinct ecological state that may reflect unique host factors or environmental selective pressures specific to this patient (Yang et al., 2023).

PICRUSt2 functional prediction indicated that metabolism-related pathways dominated the functional landscape across all genera (**Figure 9**). Carbohydrate metabolism and amino acid metabolism were the most abundant KEGG level-2 categories, followed by metabolism of cofactors and vitamins, energy metabolism, and nucleotide metabolism. These dominant metabolic functions are consistent with the nutritional demands of a diverse bacterial community inhabiting a nutrient-rich environment such as the supragingival plaque biofilm (Hernández et al., 2022). Lipopolysaccharide (LPS) biosynthesis pathways exhibited elevated predicted abundance in samples with high *Proteobacteria* content. Because LPS is a potent immune activator and a recognized driver of systemic inflammation (Maldonado et al., 2016), an enhanced LPS-producing capacity within the pre-chemotherapy oral bacteriome may, in principle, contribute to inflammatory responses during subsequent phases of neutropenia and mucosal injury (Sonis, 2004). We emphasize, however, that these functional inferences are predictive in nature and require validation through metagenomic or metatranscriptomic approaches.

Several important limitations of the present study warrant explicit acknowledgment. First, the modest sample size (n = 10) constitutes a substantial constraint that limits the generalizability of our findings and precludes robust statistical comparisons between tumor types or against reference populations. This pilot investigation was designed to generate preliminary descriptive data, and definitive conclusions must await larger, adequately powered studies. Second, the absence of a healthy pediatric control cohort precluded direct comparative analyses and constrained our capacity to distinguish disease-associated microbiome alterations from the normal inter-individual variation characteristic of oral bacterial communities during childhood (Xu et al., 2014). Third, the cross-sectional design captured the microbiome at a single time point prior to chemotherapy; consequently, the dynamic alterations occurring throughout the course of treatment remain to be elucidated in subsequent longitudinal investigations. Fourth, the unbalanced distribution of tumor types (1–3 patients per category) precludes meaningful inter-tumor comparisons, and any apparent associations between microbial features and specific malignancies should be regarded as hypothesis-generating only. Finally, functional inferences derived from PICRUSt2 and BugBase are predictive and based on 16S rRNA gene data; these predictions require validation through shotgun metagenomic or metatranscriptomic approaches to confirm the presence and activity of the inferred metabolic pathways (Douglas et al., 2020).

The baseline data presented herein establish a foundation for several avenues of future inquiry. Notably, a parallel internal transcribed spacer (ITS) sequencing analysis was performed on companion samples from the same cohort to characterize the oral mycobiome, and these findings are reported in a separate publication. Integrating the bacteriome and mycobiome datasets will provide a more comprehensive understanding of cross-kingdom microbial interactions within the oral cavity of pediatric cancer patients prior to chemotherapy (Baker et al., 2017). Longitudinal investigations tracking the oral microbiome across successive chemotherapy cycles will be essential for identifying specific taxonomic shifts associated with mucositis, infection, and treatment-related complications. Furthermore, multi-center studies incorporating larger cohorts, matched healthy controls, and standardized sampling protocols are warranted to validate these preliminary observations and to clarify the clinical relevance of baseline oral microbial profiles in predicting chemotherapy outcomes.

In conclusion, this pilot study used PacBio full-length 16S rRNA gene sequencing with DADA2-based ASV inference to characterize the oral bacteriome of pediatric cancer patients before chemotherapy. We observed a diverse but highly individualized bacterial community, with marked variability between patients in composition, diversity, network structure, and oxygen-utilization phenotypes. Beta diversity analysis showed that community composition was driven mainly by inter-individual differences rather than tumor type, with two samples (M7 and M10) standing out as distinctly divergent. These findings offer a baseline reference for future longitudinal studies of chemotherapy-induced dysbiosis. Larger studies with healthy controls, clinical outcome data, and metagenomic validation are needed to confirm these observations and to determine whether baseline oral microbiome features can serve as predictive biomarkers of treatment-related complications in pediatric oncology.

## Data Availability

The raw data supporting the conclusions of this article will be made available by the authors, without undue reservation.
